# Miscellaneous

**Published:** 1890-10

**Authors:** 


### MISCELLANEOUS.

THE lamp business may be classed as one of the great industries of the country. Within a few years a great change has taken place in the business. Glass and crockery lamps have given way to bronze, iron and brass. In Connecticut alone the following cities and populations are largely engaged in the manufacture of bronze and brass lamps: Meriden, 22,000; Waterbury, 30,000; Birmingham and Ansonia, 20,000; Bridgeport, 50,000. Total, 122,000 people. The business has increased immensely since the "Rochester" bronze lamp was introduced with its perforated cone burner, about five years ago.

### HOT WATER IN A HURRY.

Very often a physician needs hot water in the middle of the night, when there are no conveniences at the house of his patient to obtain it. Especially is this apt to be the case in summer time. If there should be a kerosene lamp handy, with a chimney having a corrugated top, this want can be easily met. Place an ordinary tin cup with a sufficient amount of water upon the top of the chimney. The corrugations let out the heated air so that the lamp will not smoke, and the water will quickly become hot. This is often a great convenience, and it is astonishing how quickly the needed hot water can be obtained. As a practical thing, it may serve a useful purpose.

DRY SKIN.—After meals, which are plain but good, a burning of the face and a dryness of the skin are experienced. Many persons who are the victims of dyspepsia, find that the face flushes and feels hot after their meals, and medical practitioners are in such cases consulted for remedies every day of their lives. In this particular instance, let me look at the form of the query, and see what light it throws upon the circumstances. "Plain, but good!" Nothing more excellent, nothing clearer, applied to clothing. Then it means strong, sober-colored, likely to wear long and well. But applied to food! Plain, but good, forsooth! Sometimes this may mean meat three times a day, with plum pudding *ad libitum;* it may mean all the good things of life, and some of the bad ones, too. The presumption is strong that there are errors of diet which need correcting, and these errors may be excessive quantity and wrong quality.

### A LAUGHTER MOVING PLANT.

IN a recent work on Arabia, mention is made of a shrub indigenous to that country, whose seeds produce an effect similar to that of laughing-gas. Its seed pods contain two or three black, bean-like seeds, of sweet taste, and of an opium flavor, whose odor is sickening and offensive. These seeds, pulverized, and taken in small doses, produce a peculiar effect, causing the individual to laugh, sing, dance, and indulge in all sorts of fantastic behavior, for an hour or more, when he falls into a deep sleep. When he wakes, he remembers nothing of what has transpired.

### REST.

When you are so tired as to feel "ready to drop," sit down, comb your hair and change your shoes. This will rest the head and feet and give new strength for the work which at house-cleaning or moving time refuses to be postponed. That lying down ten minutes will rest one much more than sitting down has to be reiterated often for the benefit of those ambitious women who sometimes scorn to rest in this way during the day time, and others who fear that it will be known to their discredit if they so indulge themselves. I once heard Mrs. Lincoln talk upon this topic, and I wish every farmer's wife might have heard the woman who has made housekeeping a study tell how to get rest enough to insure health. It was the wisdom, not of the theorist, but of one who had so nearly overworked as to have found it needful to study means of making housekeeping possible without slowly killing the housewife.—*New England Farmer.*

### DON'T SCOLD.

Mothers, don't scold. You can be firm without scolding your children ; you can reprove them for their faults ; you can punish them when necessary, but don't get into the habit of perpetually scolding them. It does them no good. They soon become so accustomed to fault finding and scolding that they pay no attention to it. Or, which often happens, they grow hardened and reckless in consequence of it. Many a naturally good disposition is ruined by constant scolding, and many a child is driven to seek evil associates because there is no peace at home. Mothers, with their many cares and perplexities, often fall into the habit unconsciously ; but it is a sad habit for them and their children. Watch yourselves, and don't indulge in this unfortunate and often unintentional manner of addressing your children. Watch even the tones of your voice, and, above all, watch your hearts ; for we have divine authority for saying that "out of the abundance of the heart the mouth speaketh."

---

It has recently been discovered that the Egyptian temple contained an apparatus much the same as the "nickle and slot machine" of modern times. A certain sum of money dropped into the apparatus through the slot, caused a small quantity of "holy water" to flow out, after which the outlet was automatically closed until the next customer dropped into it a piece of money of proper size. This is certainly another illustration of the fact that there is nothing new under the sun. The writer when in Italy, a few years ago, purchased among other ancient Roman curiosities, a safety-pin involving exactly the same principle as that of modern times, although differently constructed. We moderns are by no means so much wiser than the ancients, as we often imagine.

---

Cliff Dwellings in Morocco.—Recent discoveries have shown that cliff dwellings are found in great numbers in Morocco, which are now, and probably have been, inhabited from the time of their first construction. These dwellings in all particulars are like those found in Arizona and New Mexico, on this continent.

In the region of the mouth of the Rio de la Plata River, the Atlantic Ocean has been ascertained to be 40,236 feet or eight and three-fourths miles in depth. This is its greatest known depth. The average depth of all the oceans is from 2,000 to 2,500 fathoms, or from 12,000 to 15,000 feet.

---

Saved by Exercise.—A man at Englewood came to me about his daughter. She was low spirited and weak. I said, "What does she do ?" and he said she went five miles to school every day and carried a great strap full of books. "Does she walk ?" "What ?" "Does she walk ?" "No, she rides in a tram-car." "Get her a pair of good shoes, broad enough at least for two of her toes to touch the ground. Ugly, of course they're ugly, but they are comfortable. Let her get off the car one mile from home the first week. Rain, well let it rain ; I hope it will. Rain doesn't look half so bad when you are in it, as when you look at it through the window. Then let her try two miles the second week, and so on up to five." I met the father in two months. He said : "The aches are all gone, and we are afraid she'll eat the table-cover. Her brother has taught her boxing, and we are all afraid of her around the house. She's actually getting good looking."—*Ex.*

